# The relationship between interaural delay in binaural gap detection and sensitivity to temporal fine structure in young adults with or without musical training experience

**DOI:** 10.3389/fnins.2022.957012

**Published:** 2022-09-01

**Authors:** Yu Ding, Ming Lei, Chunmei Cao

**Affiliations:** ^1^Division of Sports Science and Physical Education, Tsinghua University, Beijing, China; ^2^Laboratory of Artificial Intelligence and Cognition, School of Tourism Sciences, Beijing International Studies University, Beijing, China

**Keywords:** temporal fine structure, binaural gap, break in interaural correlation, interaural delay, primitive auditory memory

## Abstract

Humans can detect the presence of a break in interaural correlation (BIC, also called binaural gap) even if a large interaural time delay (ITD) is introduced, which is important for detecting, recognizing, and localizing sounds in everyday environments. To investigate the relationship between interaural delay in binaural gap detection and the sensitivity of temporal fine structure (TFS), 40 young college students with normal hearing took the BIC delay threshold test, the TFS1 test (the test of monaural TFS sensitivity), and the TFS-AF test (the test of binaural TFS sensitivity). All participants were asked whether they had any musical training experience in their childhood. Results showed that the BIC delay threshold was significantly correlated with the TFS1 test (*r* =−0.426, *p* = 0.006), but not with the TFS-AF performance (*r* =−0.005, *p* = 0.997). The correlation between BIC delay threshold and monaural TFS sensitivity was observed in the non-music training group (*r* =−0.508, *p* = 0.010), but not in the music training group (*r* =−0.290, *p* = 0.295). These findings suggest that the interaural delay in binaural gap detection is related to the monaural sensitivity of TFS, this significant correlation was mainly found in young adults without musical training experience.

## Introduction

One of the benefits of having two ears is that binaural spatial cues can be obtained, as the time and intensity of the signal reaching both ears vary depending on the location of the sound source (Blauert, 1997; Schnupp et al., 2011). Extracting and integrating binaural information not only provides a basis for sound localization but is also crucial for target detection and speech recognition in complex environments (Bronkhorst, 2000; Darwin, 2006; Eramudugolla et al., 2008; Maddox and Shinn-Cunningham, 2012). Binaural information processing involves both the binaural calculation of the similarity of the acoustic details (mostly temporal fine structure, TFS) between the two ears (Huang et al., 2009; Li et al., 2009) and the monaural/binaural sensitivity (Moore, 2014) of the TFS signal.

Usually, speech signals are decomposed into several narrowband signals in the cochlea, and these narrowband signals can be considered as a relatively slow variation in amplitude over time (envelope, ENV) and the rapid oscillations with a rate close to the center frequency of the band (TFS) (Moore and Sek, 2009). There are already many measurement methods for TFS sensitivity. Among them, monaural sensitivity to TFS can be measured with the “TFS1” test (Moore and Sek, 2009; Sęk and Moore, 2012), while binaural sensitivity to TFS can be assessed using the “TFS-AF” (TFS-adaptive frequency) test (Füllgrabe et al., 2017). TFS1 and TFS-AF tests are mature behavioral measurement methods and have been used in many studies (Füllgrabe et al., 2018; Tarnowska et al., 2019).

Listeners are very sensitive to the dynamic changes in interaural correlation, such as detecting a dynamic break in interaural correlation (BIC, also called BIAC or binaural gap, a brief drop of interaural correlation from 1 to 0 and then return to 1) in a steady-state noise, showing the marked ability to temporally resolve fast changes in interaural configurations (Akeroyd and Summerfield, 1999; Boehnke et al., 2002). Introducing a change in interaural correlation does not change the monaural energy and spectrum in the sound signals, but changes the loudness (Moore, 2003) and dichotic repetition pitch (Bilsen and Goldstein, 1974) of the signals. A study based on the frequency-following responses (FFRs) of the rat auditory midbrain found that introducing a BIC causes more reduction in FFR_TFS_ than in FFR_Env_ (Wang and Li, 2015), and an earlier study also showed that the ENV is not as important as the TFS in determining the BIC detection (Boehnke et al., 2002).

Furthermore, even if a large interaural time delay (ITD) is introduced, humans can still detect the presence of BIC (Huang et al., 2009; Li et al., 2013; Liu et al., 2016). The past studies associated with judging sidedness showed that laterality cues can be discriminated at large ITD, which indirectly measured the ability to detect interaural correlated sounds (Mossop and Culling, 1998). Results of early studies have suggested that the representation of the TFS may persist for up to 9–15 ms (Cherry, 1954; Blodgett et al., 1956; Langford and Jeffress, 1964; Mossop and Culling, 1998). The preservation of the sensitivity to the BIC even when a large ITD is introduced indicates that the TFS information of noise is maintained for the time of the ITD (Huang et al., 2008). Measuring the ITD when the BIC is detectable can provide a way of investigating the temporal storage of acoustic details, which is called the “BIC delay threshold” test (Huang et al., 2009). This faithful auditory storage of TFS has been recognized as the early point in the chain of the transient auditory memory system and termed primitive auditory memory (PAM) (Li et al., 2013; Kong et al., 2015; Liu et al., 2016).

The relationship between interaural delay in binaural gap detection and sensitivity to TFS may vary in populations with different characteristics. A recent study (age range 21–65 years) found that the BIC delay threshold and TFS-AF tests were significantly correlated in a tinnitus group but not in a normal group, since binaural integration may be more difficult due to overt/covert hearing loss with aging and tinnitus (Ding et al., 2022). However, both BIC delay threshold and TFS-AF tests are binaural-based tests that are likely to be affected by monaural coding of TFS information before binaural interaction (Whiteford et al., 2017). Furthermore, many young participants had musical training in their childhood, and music training is related to both monaural sensitivity (Mishra et al., 2015) and binaural sensitivity (Bianchi et al., 2019) of TFS. Therefore, it is unclear whether the BIC delay threshold is associated with monaural/binaural sensitivity of TFS in young adults with or without musical training experience. This research focuses on the relationship between the BIC delay threshold and the monaural/binaural sensitivity of TFS, investigating whether the performance of the BIC delay threshold is related to the performance of the TFS1 or TFS-AF test, considering childhood musical training experience as a potential influencing condition.

## Materials and methods

### Participants

Forty university students (21 males and 19 females; mean age = 22.93 between 18 and 30 years) with normal hearing participated in this study. To estimate the required number of participants, we used the results from the first 20 participants rather than any independent estimate from the literature or a pilot. We noted that the correlation coefficient for them between the BIC delay threshold and TFS1 scores was 0.42. Entering this into G-power gave 39 as the number of participants required to maintain this value of correlation in the whole data set for α = 0.05, power = 0.8, and two tails (Faul et al., 2009). Their pure-tone thresholds were no more than 20 dB hearing level (HL) between 0.125 and 8 kHz (ANSI-S3.6, 2004) in each ear, and the threshold difference between the two ears in each frequency was less than 15 dB HL. All the participants gave their written consent to participate in the study and were paid a modest stipend for their participation. The study was approved by the Tsinghua University Ethics Committee.

All participants were asked whether they had any musical training experience in their childhood. The specific problems were stated as follows: Did you receive musical training (including professional instrumental or vocal training) in your childhood? What kind of music training did you have? When did you start musical training? How long did your music training last? Among them, 25 participants did not receive any musical training, and the other15 participants received musical training in their childhood (including 7 piano trainees, 3 guzheng trainees, 1 loner trainee, 1 electronic organ trainee, 1 harmonica trainee, and 2 vocal trainees). All musical trainees began before the age of 13 and the mean duration of their musical training was 5.93 ± 4.41 years.

### Apparatus and stimuli

All tests were carried out in a soundproof room where environmental noise was less than 29 dB SPL. All acoustic signals were calibrated by a sound-level meter (AUDit and System 824, Larson Davis, Provo, UT, United States), delivered by the Creative Sound Blaster (Sound Blaster X-Fi Surround 5.1 Pro, Creative Technology Ltd., Singapore), and presented to participants over two earpieces of Sennheiser HD 650 headphones. For the BIC delay threshold test, we performed a direct calibration on the generated noise. For TFS1 and TFS-AF tests, these two softwares have built-in calibration routines (Sęk and Moore, 2012), and we followed its procedure to calibrate.

All participants were tested for pure-tone hearing threshold first (125–8,000 Hz). The order of the three tests was randomized among participants. Before each test, there would be a practice phase to ensure that participants understood the experimental task (details of the practice phase are described below).

For the BIC delay threshold, 2,000 ms Gaussian wideband noises (including 30 ms rise-fall time, 60 dB SPL) were synthesized using the “randn()” function in the MATLAB (the Math Works Inc., Natick, MA, United States) at the sampling rate of 48 kHz with 16-bit resolution. The generated signals were then lowpass filtered at 10 kHz.

Two special software packages were used in this study to perform the testing of TFS1 (Sęk and Moore, 2012) and TFS-AF (Füllgrabe et al., 2017), which can be downloaded from the Internet.^1^ Most of the parameters use the default settings.

### Break in interaural correlation delay threshold test

For consistency and reproducibility, the parameters and procedures of the BIC delay threshold test have been described in detail in multiple previous studies (Huang et al., 2009; Li et al., 2009, 2013; Lei and Ding, 2021; Ding et al., 2022). There were two kinds of signals; in one presentation, the left-headphone noise was an exact copy of the right-headphone noise. In the other presentation, the temporal middle of the left-headphone noise was substituted with a randomly selected independent noise fragment with a fixed duration of 200 ms before filtering, introducing a brief break of interaural correlation, from 1 to 0 and then returning to 1. In the practice phase, participants became familiarized with binaurally presented noise either with or without the BIC. The task was to identify which of the two presentations contained the BIC. The offset-to-onset interval between the two presentations was 500 ms.

The longest ITD for BIC detection was measured using a three-up-one-down paradigm (Levitt, 1971): The ITD increased following three consecutive correct choices of the presentation containing the BIC, and decreased following one incorrect choice. The initial step size was 16 ms, which was altered by a factor of 0.5 with each reversal of direction until the minimum size of 1 ms was reached, and the longest ITD was defined as the mean ITD for the last six reversals. Visual feedback was given after each trial to indicate whether the choice was correct or not.

### Test for monaural/binaural sensitivity of temporal fine structure

TFS1 (Moore and Sek, 2009) and TFS-AF (Füllgrabe et al., 2017) each used methods described in the references. The TFS1 test involved discrimination of a harmonic complex tone (H, with a fundamental frequency, F0) and an inharmonic tone (I, all harmonics shifted upwards by ΔF). The task was a two-interval forced-choice task, and each interval contains four bursts of sound (HIHI or HHHH), the participants were required to discriminate harmonic complex tones and corresponding “frequency-shifted” tones by clicking on the appropriate box on the screen. The fundamental frequency was 200 Hz, the center frequency was 1,800 Hz, and the width of the passband was equal to F0 as the recommended value (Hopkins and Moore, 2011). The signal sound intensity was 60 dB SPL, the noise sound intensity was 45 dB SPL, and the initial change frequency was 100 Hz as the default settings (Sęk and Moore, 2012).

In the TFS-AF test, two consecutive intervals were presented on each trial, separated by 500 ms. Each interval contained four consecutive 400 ms tones, separated by 100 ms. In one interval, the IPD of all tones was always 0° (the standard), while tones with IPD = 0° are perceived as emanating from close to the center of the head. In the other interval, the first and third tones were the same while the second and fourth tones differed in their IPD by φ (the target). Participants were asked to indicate which of the two intervals contained a sequence of tones that appeared “Moving” within the head. The initial frequency for the TFS-AF test was 200 Hz, the sound intensity of the left and right ears was 30 dB SL (sensation level), and the phase difference (φ) was set to 180°.

The TFS1 and TFS-AF used the two-down-one-up (or two-up-one-down) procedure to estimate the “threshold” corresponding to 70.7% correct. It should be noted that the BIC delay threshold test used the three-up–one-down procedure, which was to be consistent with past studies and facilitate horizontal comparison with the results of past studies.

**FIGURE 1:**
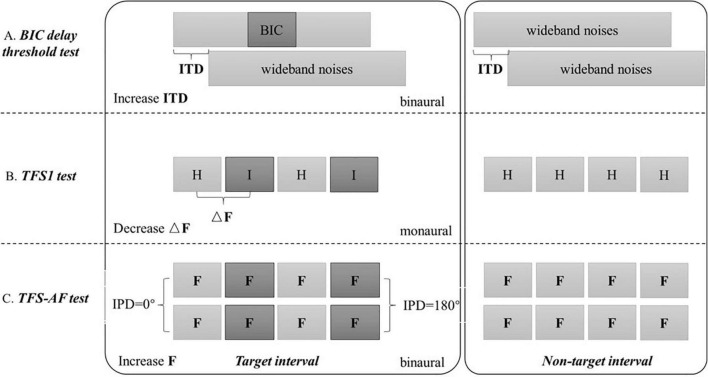
Schematic diagram of three test methods. The BIC delay threshold test, TFS1 test, and TFS-AF test use the maximum ITD, minimum ΔF/F0, and maximum F as thresholds, respectively.

All the results were automatically output by the software after the test. The principle of the three test methods used in this research is shown in Figure 1.

## Results

### Test scores of participants

The results showed that the longest ITD for the BIC detection varied between 6.5 and 18.0 ms across 40 participants (mean = 11.4 ms, SD = 3.0 ms). A previous study showed that the BIC delay threshold is related to the frequency of the noise. For narrow-band noise, the BIC delay threshold decreases with the increase of the center frequency. The BIC delay threshold is approximately 12 ms for narrowband noise (center frequency = 200 Hz) and approximately 10 ms for wideband noise (Li et al., 2013). For monaural sensitivity, the results of TFS1 showed that the relative frequency shift threshold was between 0.017 and 0.221 (mean = 0.087, SD = 0.042) for left ear, and from 0.037 to 0.152 (mean = 0.076, SD = 0.031) for right ear. The mean monaural sensitivity of both ears ranged from 0.030 to 0.170 (mean = 0.0,815, SD = 0.031). A previous study (center frequency = 2,000 Hz, F0 = 222 Hz) showed that the relative frequency shift threshold for musicians was around 0.07–0.11, and slightly higher for non-musicians, around 0.11–0.17 (Mishra et al., 2015). For binaural sensitivity, the results showed that the TFS-AF threshold varied between 1,070.6 and 2,010.0 Hz (mean = 1,359.7 Hz, SD = 193.4 Hz). A previous study showed that the threshold for TFS-AF (180°) was approximately between 1,000 and 2,000 Hz, with a mean of 1,382 Hz (Füllgrabe et al., 2017). All the above results were not far from the scope of previous reports, and all three tests varied remarkably across participants. K-S (Kolmogorov-Smirnov) tests showed that there is no evidence that any of test indicators data differ from normal distribution (for TFS1: *p* = 0.442; for TFS-AF: *p* = 0.237; for BIC: *p* = 0.884).

### The relationship of temporal fine structure sensitivities with break in interaural correlation delay threshold

**FIGURE 2:**
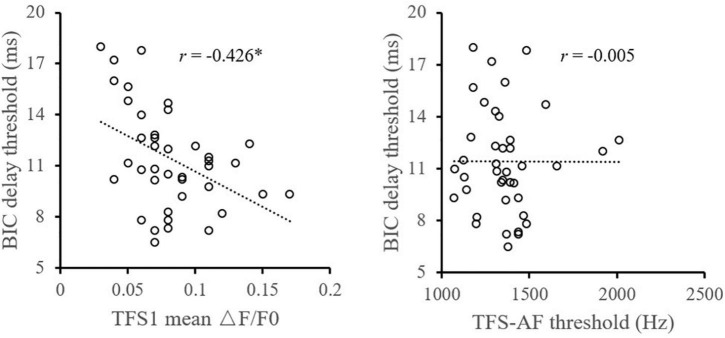
Illustration of the correlation analysis of the BIC delay threshold and the TFS1 and TFS-AF test. *Significant effect after Bonferroni’s correction, *p* < 0.025.

**FIGURE 3:**
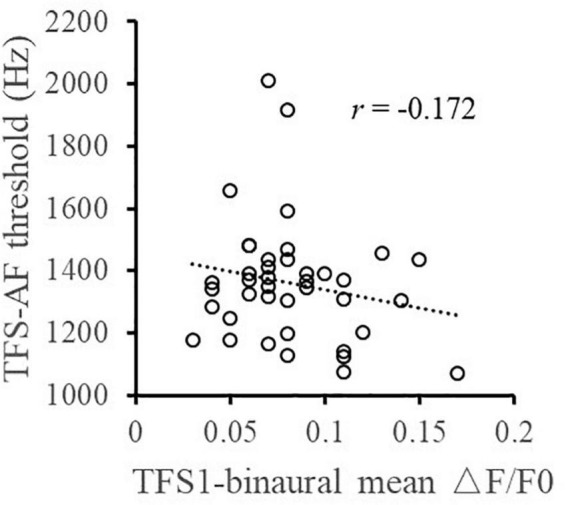
Illustration of the correlation analysis of the TFS1 and TFS-AF test.

Pearson correlation analysis (Figure 2) showed that the TFS1 score was significantly correlated with the BIC delay threshold. The monaural TFS sensitivity averaged across ears of TFS1 was significantly correlated with the BIC delay threshold (*r* = −0.426, *p* = 0.006), but there was no evidence of a significant correlation between the BIC delay threshold and TFS-AF performance (*r* = −0.005, *p* = 0.997). In addition, this study did not observe significant correlation between TFS1 and TFS-AF (*r* = −0.172, *p* = 0.289) (Figure 3).

**FIGURE 4:**
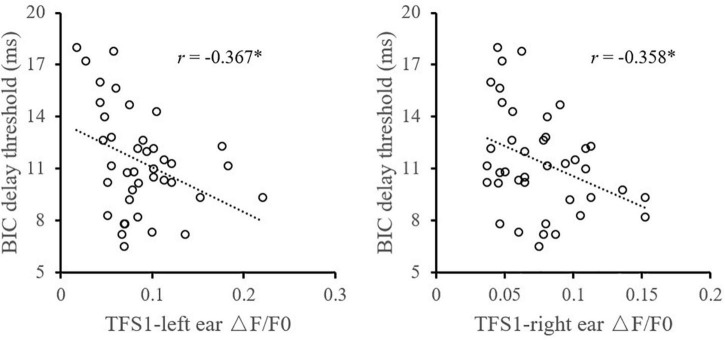
Illustration of the correlation analysis of the BIC delay threshold and the TFS1 and TFS-AF test. *Significant effect after Bonferroni’s correction, *p* < 0.025.

**FIGURE 5:**
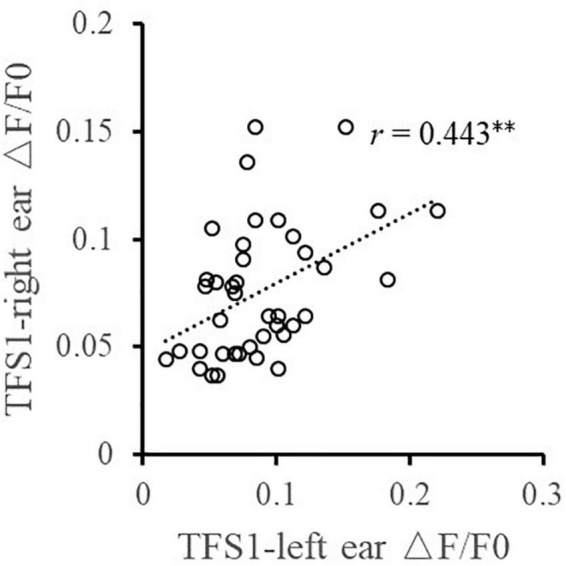
Illustration of the correlation analysis of the TFS1 scores of the left and right ears. ^**^p < 0.01.

Furthermore, the relationship between the TFS sensitivity of the left and right ears and the BIC delay threshold was investigated. Pearson correlation analysis (Figure 4) showed that the TFS1 score of the left and right ears was significantly correlated with the BIC delay threshold (for left ear: *r* = −0.367, *p* = 0.020; for right ear: *r* = −0.358, *p* = 0.023). Figure 5 shows that there was a significant correlation between TFS1 scores of the left and right ears (*r* = 0.443, *p* = 0.004).

### The effect of music training

**FIGURE 6:**
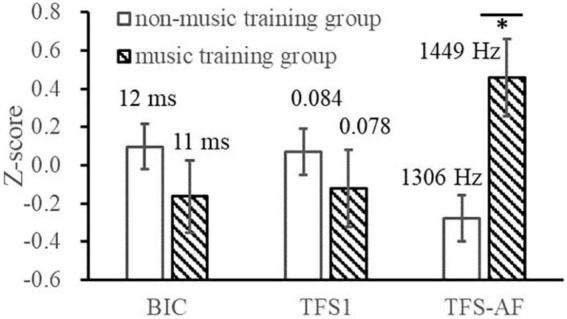
Illustration of the BIC delay threshold and TFS sensitivities of the music training group and the non-music training group. *p < 0.05.

This study investigated whether the music training would affect the measurement results (and their relationship) of the BIC delay threshold and TFS sensitivity tests (Figure 6). A 3 (Test indicators: BIC delay threshold, TFS1 binaural mean, TFS-AF threshold) × 2 (music training experience: music training group, non-music training group) within-subject repeated measures ANOVA showed that the interaction between the two factors was significant [*F*(2, 76) = 5.729, *p* = 0.005, η*_*p*_*^2^ = 0.131] and the main effect of the music training experience was significant [*F*(1, 38) = 5.623, *p* = 0.023, η*_*p*_*^2^ = 0.129]. The independent sample *t*-test showed that the music training group had better TFS-AF scores [1,448.7 Hz for the music training group and 1,306.2 Hz for the non-music training group, *t*(38) = 2.386, *p* = 0.022]. Note that for the TFS-AF and BIC delay threshold tests, higher scores are better, while for the TFS1 test, lower scores represent higher sensitivity. Therefore, it indicates that for musical training experience, both monaural and binaural showed a trend toward better sensitivity to TFS, while the BIC delay threshold did not. It is also important to note that this study did not specifically recruit music majors, but only considered and recorded the effects of music training in normal participant recruitment. Such surveys lack necessary information, such as music level and daily training duration, so this grouping is insufficient compared to the definition of musicians in previous studies and leads to a reduction in statistical power. Insufficient statistical power means that there is a greater chance of making Type 2 errors (β), and some effects may not be detected. Therefore, some interpretations of the results need to be conservative. We used the G-power software to calculate the t-tests achieved power (*post hoc*) of the BIC delay threshold, TFS1, and TFS-AF, which were 0.09, 0.17, and 0.59, respectively (Faul et al., 2009). It should be noted that a reduction in statistical power may affect the reproducibility of this part of the results.

**FIGURE 7:**
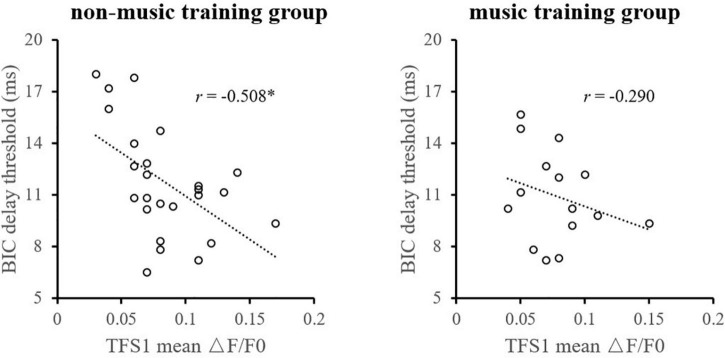
Illustration of the correlation analysis of the BIC delay threshold and the TFS1 and TFS-AF test. *Significant effect after Bonferroni’s correction, *p* < 0.025.

Considering the influence of music training experience, the relationship between TFS1 and BIC was compared between the music training group and those without any music training experience, respectively (Figure 7). For participants without any musical training experience, the monaural TFS sensitivity averaged across ears of TFS1 is significantly correlated with the BIC delay threshold (*r* = −0.508, *p* = 0.010). For participants with musical training experience, there was no evidence of a significant correlation between the BIC delay threshold and TFS1 performance (*r* = −0.290, *p* = 0.295).

## Discussion

The results of this study found that, in young adults, the maximal ITD of detecting binaural BIC was significantly correlated with monaural TFS sensitivity, but not with binaural TFS sensitivity. The correlation between BIC delay threshold and monaural TFS sensitivity was observed in both left and right ears, but this correlation was not found in the participants with musical training experience.

Binaural information integration is crucial for speech recognition in complex scenes. In previous studies, the BIC delay threshold was considered an effective method to measure transient the auditory storage capacity of acoustic details (Li et al., 2013; Kong et al., 2015; Liu et al., 2016). However, by measuring and comparing the effects of interaural delay and interaural correlation in a group of participants, a previous study discovered a linear relationship between the changes in interaural correlation and interaural delay required to produce an equivalent decline of sensitivity to the BIC: an increment of 1 ms in BIC delay threshold is equivalent to a reduction of about 0.07 in interaural correlation (Kong et al., 2015). Furthermore, BIC detection involves not only the binaural calculation of the similarity of the TFS signals between the two ears but also the monaural coding of the TFS signal (Lei and Ding, 2021). Although introducing a change in interaural correlation does not alter the monaural energy spectrum of the sound signals, it changes dichotic repetition pitch (Bilsen and Goldstein, 1974) and the loudness (Moore, 2003) of the noise. Therefore, the detection ability of BIC is related to the sensitivity of pitch and loudness, while the TFS1 test reflects the pitch sensitivity to a certain extent, which may be one of the reasons why the two tests are related. In summary, the BIC delay threshold test primarily examines the ability to temporally store sound details, but it also reflects sensitivity to changes in interaural correlation and is associated with many monaural sensitivities.

Music training is related to both monaural sensitivity (Mishra et al., 2015) and binaural sensitivity (Bianchi et al., 2019) of TFS. Studies have found that compared to non-musicians, musicians have a superior ability to discriminate complex sounds based on their TFS, and this ability is unaffected by contralateral stimulation or ear of presentation (Tarnowska et al., 2019). Our study faced the problem of being underpowered (sample sizes: 15 with training, 25 without) but showed similar trends. Studies on BIC testing for musicians are lacking, and no significant results were observed in this study. BIC delay threshold and TFS1 test scores were only significantly correlated in the non-music training group, which may be due to the different effects of music training on those abilities, such as improving TFS sensitivity. This suggests the importance of background checks on participants in auditory-related research, considering that there may be many people who have received musical training in their childhood and that even non-professional training may have an impact on the test results.

## Summary

Overall, the measurements did not show any significant link between the BIC delay threshold and binaural TFS sensitivity, though we note the experimental power was low. However, test scores showed that the BIC delay threshold was significantly correlated with monaural TFS sensitivity. The significant correlation between the BIC delay threshold and monaural TFS sensitivity was mainly found in young adults without musical training experience.

## Data availability statement

The raw data supporting the conclusions of this article will be made available by the authors, without undue reservation.

## Ethics statement

The studies involving human participants were reviewed and approved by Tsinghua University Ethics Committee. The patients/participants provided their written informed consent to participate in this study.

## Author contributions

YD and ML: conception, design, acquisition of data, analysis of data, interpretation of data, and writing – original draft and review and editing. CC: conception, design, funding acquisition, and writing—review and editing. All authors contributed to the article and approved the submitted version.

## Footnotes

1
https://www.psychol.cam.ac.uk/hearing
